# Aquaculture: global status and trends

**DOI:** 10.1098/rstb.2010.0170

**Published:** 2010-09-27

**Authors:** John Bostock, Brendan McAndrew, Randolph Richards, Kim Jauncey, Trevor Telfer, Kai Lorenzen, David Little, Lindsay Ross, Neil Handisyde, Iain Gatward, Richard Corner

**Affiliations:** 1 Institute of Aquaculture, University of Stirling, Stirling FK9 4LA, UK; 2 Division of Biology, Imperial CollegeLondon, Ascot, UK

**Keywords:** aquaculture, resources, integration, development, competitiveness

## Abstract

Aquaculture contributed 43 per cent of aquatic animal food for human consumption in 2007 (e.g. fish, crustaceans and molluscs, but excluding mammals, reptiles and aquatic plants) and is expected to grow further to meet the future demand. It is very diverse and, contrary to many perceptions, dominated by shellfish and herbivorous and omnivorous pond fish either entirely or partly utilizing natural productivity. The rapid growth in the production of carnivorous species such as salmon, shrimp and catfish has been driven by globalizing trade and favourable economics of larger scale intensive farming. Most aquaculture systems rely on low/uncosted environmental goods and services, so a critical issue for the future is whether these are brought into company accounts and the consequent effects this would have on production economics. Failing that, increased competition for natural resources will force governments to allocate strategically or leave the market to determine their use depending on activities that can extract the highest value. Further uncertainties include the impact of climate change, future fisheries supplies (for competition and feed supply), practical limits in terms of scale and in the economics of integration and the development and acceptability of new bio-engineering technologies.

In the medium term, increased output is likely to require expansion in new environments, further intensification and efficiency gains for more sustainable and cost-effective production. The trend towards enhanced intensive systems with key monocultures remains strong and, at least for the foreseeable future, will be a significant contributor to future supplies. Dependence on external feeds (including fish), water and energy are key issues. Some new species will enter production and policies that support the reduction of resource footprints and improve integration could lead to new developments as well as reversing decline in some more traditional systems.

## 1. The aquaculture sector: key features and trends

### (a) Output, value and regional overview

#### (i) Current status

**Figure 1.**
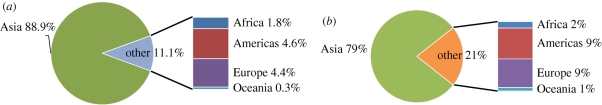
Global aquaculture production by region. Source: FAO (2010). (*a*) Aquaculture by quantity 2008 (excluding aquatic plants). (*b*) Aquaculture by value 2008 (excluding aquatic plants). Dark blue, Africa; brown, Americas; light green, Asia; violet, Europe; light blue, Oceania.

Global aquaculture (figure 1) has grown dramatically over the past 50 years to around 52.5 million tonnes (68.3 million including aquatic plants) in 2008 worth US$98.5 billion (US$106 billion including aquatic plants) and accounting for around 50 per cent of the world's fish food supply. Asia dominates this production, accounting for 89 per cent by volume and 79 per cent by value, with China by far the largest producer (32.7 million tonnes in 2008). The rapid growth in this region has been driven by a variety of factors, including pre-existing aquaculture practices, population and economic growth, relaxed regulatory framework and expanding export opportunities.

Aquaculture development in Europe and North America was rapid during the 1980s–1990s but has since stagnated, probably owing to regulatory restrictions on sites and other competitive factors, although as markets for fish and seafood they have continued to grow.

#### (ii) Growth rates

The growth rate of aquaculture between 1970 and 2006 was 6.9 per cent per annum (FAO 2009*a*), although it appears to be slowing (average 5.8% between 2004 and 2008). This reflects the typical pattern, which can be seen at the national level, of adoption followed by rapid growth, which then slows with increasing competition and other constraints.

**Figure 2.**
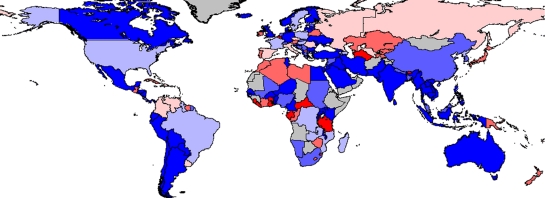
Average annual growth rate of all aquaculture production in terms of quantity over a 5-year period. Calculated using the difference between mean values from the periods 2000–2002 and 2005–2007. Red, greater than −10%; orange, −3 to −10%; rose, 0 to −3%; violet, 0–3%; light blue, 3–10%; dark blue, greater than 10%. Source: FAO (2009*b*).

**Figure 3.**
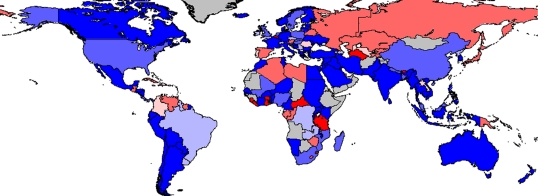
Average annual growth rate of all aquaculture production in terms of value over a 5-year period. Calculated using the difference between mean values from the periods 2000–2002 and 2005–2007. Red, greater than −10%; orange, −3 to −10%; rose, 0 to −3%; violet, 0–3%; light blue, 3–10%; dark blue, greater than 10%. Source: FAO (2009*b*).

The highest relative growth rates between 2006 and 2007 are in countries with relatively low production, such as Lesotho (6450%), Rwanda (909.5%) and Ukraine (590.8%). Although these can be a useful indicator of new initiatives, smaller percentage growth in countries with already substantial production has a greater impact. For instance, 5.2 per cent growth in China represented 52.3 per cent of the total increase in global aquaculture supply for 2007. The second most important country in this respect was Vietnam, which contributed 16.7 per cent of the additional aquaculture production with a growth rate of 30.1 per cent (figures 2 and 3).

A small number of countries with substantive production experienced contraction in 2007, most notably Thailand, Spain and Canada. Reasons for this were mainly market and competitiveness related, although disease and one-off environmental events can also play a role in single-year figures. Overall, these reductions amounted to the equivalent of 1.6 per cent of global supplies (i.e. more than compensated by growth elsewhere).

#### (iii) Species produced

Excluding aquatic plants, 310 species were recorded by FAO as cultured in 2008. However, the top five species accounted for around 33 per cent of the output (19% by value), the top 10 for 53 per cent (45% by value) and the top 20 species for 74 per cent of production by volume (63% by value). Freshwater fish production is dominated by various species of carp, although tilapia and later pangasius catfish have become more significant (table 1). Coastal aquaculture primarily comprises whiteleg and, to a lesser extent, tiger shrimp, oyster, scallop and mussels, with Atlantic salmon as the leading intensively farmed marine fish.

**Table 1.** Most significant species (more than 1 million tonnes in 2008), by quantity and value. Developed from Fishstat data FAO (2010). nei, not elsewhere included.

	number of countries	output (tonnes)	% change		value, '000 US$	US$ per kg	% change	
		2008	1 year	10 years mean	2008	2008	1 year	10 years
silver carp	50	3 848 258	5.5	2.8	4 864 708.1	1.26	13.4	8.5
grass carp (=white amur)	57	3 775 267	4.3	4.0	4 797 278.6	1.27	12.2	9.5
cupped oysters nei	18	3 385 382	−4.5	3.1	2 023 425.8	0.60	7.8	−1.3
Japanese carpet shell	13	3 141 851	3.2	13.4	3 185 467.0	1.01	14.7	7.7
common carp	100	3 000 529	5.5	3.7	3 758 752.0	1.25	7.2	6.7
Nile tilapia	74	2 334 432	8.6	22.2	3 208 560.8	1.37	8.2	27.5
bighead carp	28	2 321 513	7.2	6.3	2 975 411.8	1.28	14.2	13.0
catla	5	2 281 838	7.9	31.1	3 303 123.8	1.45	11.4	49.7
whiteleg shrimp	36	2 259 183	−1.7	106.7	8 985 288.8	3.98	−0.8	78.1
Atlantic salmon	31	1 456 721	5.6	11.2	7 204 151.8	1.44	1.0	23.6
pangas catfishes nei	5	1 380 702	52.4	220.1	1 994 685.4	1.43	49.1	197.8
freshwater fishes nei	97	1 247 859	−25.1	−1.2	1 778 408.2	1.15	−29.1	1.6
roho labeo	6	1 159 454	19.6	7.6	1 334 192.7	1.42	20.1	−0.5
scallops nei	7	1 137 379	−2.4	9.9	1 615 936.3	1.26	9.1	10.2

### (b) Aquaculture systems and environments

#### (i) Freshwater ponds and tanks

**Figure 4.**
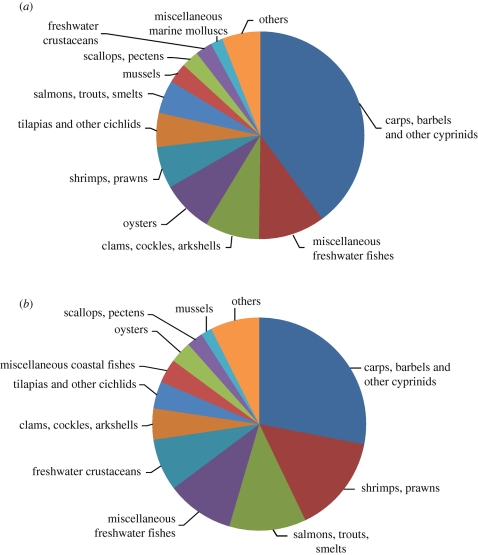
Aquaculture production by output and value for major species groups in 2008. Source: FAO (2010), excluding aquatic plants. (*a*) Aquaculture by output 2008 (excluding aquatic plants). (*b*) Aquaculture by value 2008 (excluding plants).

Freshwaters were the source for 60 per cent of the world aquaculture production in 2008 (56% by value), despite they only constituting 3 per cent of the planet's waters and only 0.3 per cent of that being surface water (figure 4). Of this, 65.9 per cent were carp and other cyprinids which are mostly cultured in ponds using semi-intensive methods (water fertilization with inorganic and organic fertilizers and supplementary feeding with low-protein materials). Salmonid farming (mainly rainbow trout in freshwater) constituted only 1.5 per cent, typically using ponds, concrete raceways and other types of tank that require higher throughputs of water to maintain a good water quality. Stocking densities are typically two to five times as high as in semi-intensive ponds and fully formulated diets are fed. Species such as tilapia (7.6% of freshwater production) are cultured in a mix of systems, from extensive to highly intensive.

#### (ii) Freshwater cages

Cage-based aquaculture in both freshwater lakes and rivers has flourished in many countries, although some are now regulating use due to concerns over environmental impacts. In Egypt, over 10 per cent of freshwater aquaculture production in 2005 was from cages in the River Nile. However, by 2006, almost 80 per cent were removed (down from 12 495 to 2702). Rapid expansion of cage-based catfish farming in the Mekong is giving similar cause for concern, but has not led to such a drastic regulatory response, although the expansion of pond farms is now apparent. In unregulated conditions, eutrophication from cage farms can impact on farms downstream, on other water uses and ecosystems in general.

**Figure 5.**
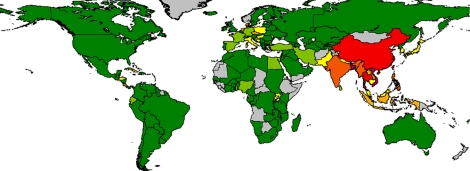
Mean aquaculture production from freshwater systems as a function of land area (kg km^−2^ yr^−1^) for the period 2005–2007. Dark green, 0–50 kg km^−2^; light green, 50–100 kg km^−2^; yellow, 100–250 kg km^−2^; light orange, 250–500 kg km^−2^; dark orange, 500–1000 kg km^−2^; red, 1000–3000 kg km^−2^; maroon, greater than 3000 kg km^−2^. Source: FAO (2009*b*).

Globally, Asia and especially China has the greatest freshwater aquaculture production in relation to land area, although some European and African countries are also significant (figure 5). The Americas in particular are notable for relatively low freshwater aquaculture production per unit area.

#### (iii) Coastal ponds and tanks

**Figure 6.**
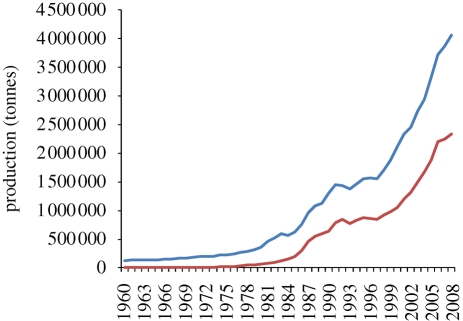
Total world production for culture of brackish-water species (blue) and for penaied shrimp (red). Source FAO (2010).

Coastal ponds and lagoons and have been exploited in simple ways for fish, mollusc, crustacean and seaweed production for centuries. However, production has been expanded and intensified over the past 30 years. In warmer countries, the penaeid shrimps have tended to dominate brackish-water culture due to high-value, short production cycles and accessible technologies. Production has increased almost exponentially since the mid-1970s (figure 6) and now accounts for about 58 per cent of aquaculture production from brackish water (72% by value).^1^ In more temperate climates, brackish-water fish species are the main crop with varying degrees of intensification.

Further expansion of brackish-water aquaculture is possible, especially in relatively unexploited regions of Africa and Latin America. However, a strengthening environmental lobby as well as competition for land resources in some areas is likely to limit developments of the kind seen in some Asian countries.

Coastal aquaculture using onshore tanks has developed in some areas (e.g. South Korea, Spain, Iceland), usually where other types of aquaculture would not be possible. Most use pumped water that passes through the tanks once before being discharged to the environment. However, an increasing number treat and reuse the water flow, providing greater isolation from the environment and hence biosecurity.

#### (iv) Coastal cage farms

**Figure 7.**
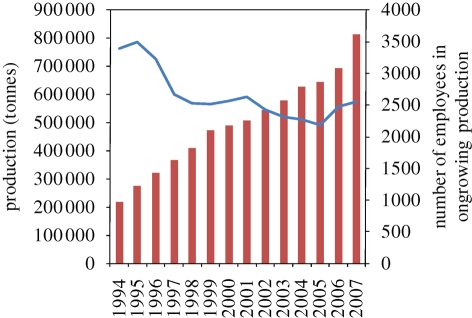
Development of production volume of Atlantic salmon and rainbow trout in Norway and number of employees (blue), illustrating trends in industrialization of production (red) systems. Source: Fiskeridirektoratet (2008).

For marine fish species with mid to high-value, floating cages have proved the most cost-effective production system across a range of farm sizes and environments (as determined by conventional financial appraisal; Halwart *et al.* 2007). The open exchange of water through the nets replenishes oxygen and removes dissolved and solid wastes. Most rely on feeding either complete diets or, for some species, trash fish. Cage units can be sized and arranged flexibly to meet the needs of the farm. Expansion is straightforward by increasing cage volume or number of units. Larger cages, especially in more exposed locations, become difficult and costly to manage with manual labour, so a range of specialist service vessels and equipment has been developed, especially in the salmon sector, to overcome such constraints (figure 7). Economies of scale supported by mechanization have helped to reduce production costs substantially.

#### (v) Marine molluscs and aquatic plants

The cultivation of marine molluscs (mainly bivalves) and seaweed using simple methods has a long history in many countries and has become widely established as a coastal livelihood activity involving high labour inputs. Since the 1990s, however, there has been significant upscaling of production and the introduction of specialized equipment allowing larger sites and greater labour efficiencies. Total output of molluscs from coastal waters in 2008 was 12.8 million tonnes valued at US$12.8 billion. A further 15.7 million tonnes of seaweeds were cultivated in coastal waters in 2008 valued at US$7.4 billion.

## 2. Resource and development interactions

### (a) Factors in aquaculture development

The development of aquaculture depends on the interaction of a wide variety of factors as summarized in table 2.

**Table 2.** Factors in the development of the aquaculture sector. Developed from Muir & Young (1998).

factor	implications
market demand	good demand and high prices for selected species in traditional markets offering initial targets for producers; steadily growing developed markets for major species (market competitiveness a central factor in shaping viable production systems)
environments	initial availability of inland waters, lagoons, sheltered bays, with suitable water quality, production temperatures, nutrient supply for shellfish and other systems (influenced by consideration of environmental impacts, conflicts or synergies with other resource users and policy with respect to land and water area ownership or rental)
infrastructure	available or improving transport, power, communications, access to major markets, good information system; scientific support structure
technical capability	emerging and rapidly establishing techniques for hatchery production, husbandry, feeds, ponds, cage and other culture systems; improvements to traditional systems, opportunities for integration with other activities and sectors
investment	local, national and regional private, commercial and institutional investment; incentives and support schemes for development, and technical research
human resources	initial nucleus of primary technical skills, developed through pioneer companies and development centres; increasing level of management skills in core groups
institutional system	generally positive and proactive environment, providing strategic research inputs, adapting to changing needs of industry, development of legal and regulatory systems

It is instructive to study individual aquaculture industries in relation to these factors. The primary factors are market demand (and competition), the availability of environmental resource, the development or transfer of appropriate technology and a favourable business environment that allows entrepreneurs to profit from their investment in the sector. However, there are many examples of failure of development, especially in Africa and parts of Latin America, due to the lack of well-developed markets or the ability to reach them due to infrastructure issues, including the lack of adequate quality controls for export. Weak institutional systems and lack of investment have also been important constraints in many countries.

### (b) Sector participants

The aquaculture sector overall is highly diverse and fragmented, ranging from smallholder ponds in Africa providing a few kilos of fish per year to international companies with annual turnover in excess of US$1 billion. An estimated 9 million people were engaged in fish farming in 2006 (FAO 2009*a*), around 94 per cent in Asia. Average output per person per year was 5.96 tonnes, but this varied from 0.57 tonnes in Indonesia, where aquaculture systems tend to be labour intensive, to 161.22 tonnes in Norway, which is highly industrialized (table 3).

**Table 3.** Examples of aquaculture employment, output and value. Adapted from FAO (2009*a*).

location	employment, '000		growth % yr^−1^	aquaculture, 2006		output, tonnes per pers	value, US$ per pers
	2000	2006		million tonnes	billion US$		
world	7671.6	8662.6	2	51.65	78.76	5.96	9092
China	3722.3	4502.8	3.2	34.43	38.42	7.65	8532
Indonesia	2142.8	2275.0	1	1.29	2.25	0.57	989
Norway	4.6	4.4	−0.8	0.71	2.72	161.22	617 620

For many participants, aquaculture is one of a more limited range of economic activities available in the specific coastal or rural location and is particularly important in countries such as Bangladesh, India and Vietnam, as both subsistence and cash crop. The number of small–medium enterprises and sole traders in Europe is also high, with 13 139 companies with an average of 2.6 full-time employees and turnover of around €270 000 (Framian 2009). However, trends towards industrialization and consolidation are strong for some species, especially commodity products that are internationally marketed. For instance, four companies now account for 70 per cent of Scottish salmon production and two for over 50 per cent of industry value.

There are critical linkages between market chain structure and viable company size. In Europe, the smallest companies tend to market directly to consumers and local hotels and restaurants, gaining a valuable premium on normal wholesale market prices. This is not an option for slightly larger producers who would saturate local markets. Scale economies become more important when producers are competing in larger markets and when there are minimum purchase quantities imposed by much larger buyers. The formation of producer cooperatives has sometimes enabled smaller companies to work with more consolidated market chains, most frequently when consolidation of sites is not physically possible.

International market chains are also impacting on previously small-scale producers in Asia. For instance, consolidation is apparent in the Vietnamese catfish industry, mainly driven by the implementation of western quality standards, initially in processing, but increasingly stretching into production. Elsewhere in Asia, complex chains involving many small companies still exist. Efficiency comes through specialization and competition on flexibility and quality of service. A key example of this is the production of live marine fish for restaurants and specialist markets in Korea, Hong Kong, China and other parts of Southeast Asia where values are very different from those of the Western markets.

### (c) Resource interactions and dependencies

#### (i) Land water and energy

While beneficial in many ways, the growth of aquaculture is increasing pressure on natural resource inputs, notably water, energy and feed, although sites in a broader sense are also an issue. There is also the question of the use of, and impact on, environmental services, particularly for the dispersion and treatment of farm effluents. Aquaculture systems are very diverse with respect to their dependence on these resources (table 4).

**Table 4.** Typical aquaculture resource demands by species. Sources: Muir & Beveridge (1987), Phillips *et al*. (1991), EIFAC (2000), FAO (2000), Green & Engle (2000), Troell *et al*. (2004) and Tyedmers *et al*. (2007). Protein energy per tonne for all fish/shellfish species = 4.73 GJ; for aquatic plants = 3.55 GJ.

	production per unit area (land or water) t ha^−1^	water use per unit of production '000 m^3^ tonne^−1^	input : output energy ratio	system features
salmon, trout and other salmonids	1750	2260^a^	50	intensively fed cage/ponds
sea bass, bream and similar	1125	2500	40	intensively fed cages
halibut, turbot, sole, etc.	2676	2000	45	intensive onshore tanks
cod, haddock, hake, etc.	1200	2500	45	experimental cage systems
carp, tilapia, catfish	2	5^a^	30	fertilized ponds
eels, sturgeon, perch, zander, etc.	190	0.1^a^	35	extensive stocked water bodies
tuna	300	3000	50	intensively fed cages
mussels	76	3000	10	raft or longline systems
oysters and scallops	25	2000	5	rafts or longlines—lanterns
clams, cockles, etc.	0.5	2000	5	extensive coastal beds
new non-fish aquaculture sp.	150	0.2	20	range of systems
aquatic plants	1	2000	1	coastal beds/stakes and lines

^a^Water consumption is mainly of concern in freshwater systems (the category salmon and trout covers a mix of both freshwater and sea water). These figures contrast with those of Verdegem & Bosma (2009) who estimated total water withdrawal for freshwater aquaculture at 16 900 m^3^ tonne^−1^, although this does not take account of water returned to the aquifer.

Freshwater farming uses a range of systems, from static water ponds through to high flow-through tanks. Most involve intake of water from the environment and a post-production effluent stream, so that water consumption does not equate to water intake. However, the quality of discharge water is usually diminished and water can be lost through evaporation and seepage. As a worst case, pond systems in tropical countries can lose 20 per cent of their volume per day (Beveridge 2004). However, pond aquaculture can also contribute to water management as it acts to catch and store surface water (rain and run-off) that might otherwise be lost from local agroecosystems or which might cause damaging floods (e.g. in the Czech Republic). Implementation of water reuse and recirculation systems can reduce consumption substantially, although usually at the cost of higher energy inputs.

The majority of freshwater aquaculture is pond based using semi-intensive methods that rely on controlled eutrophication for their productivity, using a wide variety of organic and inorganic fertilizers as well as supplementary feedstuffs. The production of feed materials for aquaculture, particularly grain and similar crops, incurs additional freshwater use (up to 3000 m^3^ tonne^−1^ according to Verdegem & Bosma 2009). Solid wastes produced from such systems often have a use as fertilizers for other crops. Dissolved nutrients can often be lost through necessary water replacement regimes and sometimes cause problems in areas with extensive aquaculture production or with otherwise oligotroiphic or mesotrophic environments. Better optimization of freshwater production systems with respect to water and feed management could triple production without increasing freshwater usage according to Verdegem & Bosma (2009).

Given the presently increasing pressures on freshwater supplies, future aquaculture development might be expected to utilize more abundant brackish and sea water resources. However, environmental issues are no less complex.

#### (ii) Productivity in relation to energy inputs

The energy cost and linked implications for carbon emissions of aquaculture activities is receiving greater attention. A distinction needs to be drawn in analysis between direct energy use (e.g. fuel and electricity consumed directly in the production process) and the more comprehensive approaches to auditing energy inputs. For instance, these may include consideration of industrial energy (energy used in the manufacture and supply of equipment, feeds and other inputs) or embodied energy, which also takes into account photosynthesis and sunlight energy or calorific values, etc. Another consideration is whether the energy sources are renewable or not.

Life cycle analysis (LCA) carried out by Tyedmers & Pelletier (2007) found energy dependence correlated with production intensity. This is mainly due to the energy input in the production and delivery of feed (Grönroos *et al*. 2006). More variable is the energy required for other on-farm activities which can range from virtually zero up to about 3 kWh kg^−1^. For land-based farms, most of the power is likely to be provided by electricity from the central grid. Cage-based farms rely mainly on diesel or other fossil fuel. Table 5 shows typical embodied energy levels and ratios for different production systems, with seaweed and mussel culture requiring much more modest input levels.

**Table 5.** Total embodied energy relationships, for equivalent area. Developed from Muir (2005), Troell *et al*. (2004) and Tyedmers *et al*. (2007).

quantity	seaweed culture	mussel culture	cage salmonid culture
energy inputs (kcal × 10^5^)			
solar/renewable (%)	0.30 (4.5%)	0.75–2.05 (71.4–85.4%)	470–830 (81.0–87.4%)
fossil/non-renewable (%)	6.35 (95.5%)	0.30–0.35 (28.6–14.6%)	110–120 (19.0–12.6%)
total energy	6.65	1.05–2.40	580–950
protein output (kcal)	6605	255–440	22 420
input/output ratio	100	410–545	2585–4235

#### (iii) Coastal zone pressures and ecosystem impacts

**Figure 8.**
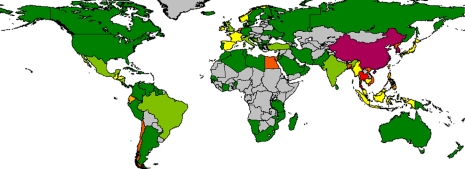
Mean production quantities from coastal aquaculture systems as function of coastline length (kg km^−1^ yr^−1^) for the period 2005–2007. Dark green, less than 10 kg km^−1^ yr^−1^; light green, 10–25 kg km^−1^ yr^−1^; yellow, 25–50 kg km^−1^ yr^−1^; light orange, 50–100 kg km^−1^ yr^−1^; dark orange, 100–250 kg km^−1^ yr^−1^; red, 250–500 kg km^−1^ yr^−1^; maroon, greater than 500 kg km^−1^ yr^−1^. Source: FAO (2009*b*).

Aquaculture, especially in coastal zones, is frequently in competition with other uses of the resource that can often take precedence (e.g. tourism and port developments; figure 8). However, there are also cases where aquaculture has outcompeted other users, such as shrimp farming, which has come under scrutiny due to over-exploitation and destruction of mangrove resources, as well as other environmental impacts and serious disease problems. The wider ecosystem value of these environments is now recognized and suitable protection given in most regions, although much remains to be done with respect to rebuilding lost area. More recent moves by the shrimp industry inland have also caused problems with saline intrusion into agricultural soils.

The development of marine fish farming in cages has also raised concerns over wider environmental, ecosystem and biodiversity impacts. At modest scales of development, these are hard to detect apart from localized changes to sediments beneath the cages. Larger scale development has the potential for wider impacts due to the release of nutrient or chemical wastes directly into the environment, or the effects of escaped fish or disease transfer on wild populations.

The most immediate problem is often conflicts between cage-based farming and other interests, such as boating and navigation, recreation, preservation of seascape scenery and protection of wildlife. In Europe, these issues are considered during the licensing process or increasingly through the development of coastal zone plans. Similar issues apply to coastal pond and pump-ashore tank systems. Recirculated water systems overcome a number of these constraints, but except for more specialist applications have so far been unable to compete financially.

#### (iv) Feeds

Most mollusc culture requires no feed inputs and the majority of freshwater fish production utilizes low-protein, grain-based supplementary diets and organic fertilizers. Much of the crustacean farming, most marine species and other intensive fish aquaculture require a higher quality diet, usually containing fish meal and often fish oil. Some aquaculture, notably tuna fattening and much of the marine cage culture in Asia, relies directly on wild-caught small pelagic fish with relatively low market price. The process transforms fish protein from low to high value for human consumption. However, the efficiency of this is both an ecological issue and one of social justice (e.g. consumers of farmed salmon and shrimp may effectively outcompete rural poor for this fish resource; Tacon & Metian 2009). Fish meal has also traditionally been used in intensive livestock rearing, especially pork and poultry, so the issues are not unique to aquaculture. However, it is aquaculture that is taking a growing and majority share of this resource as substitutes are more easily found for livestock and poultry.

**Figure 9.**
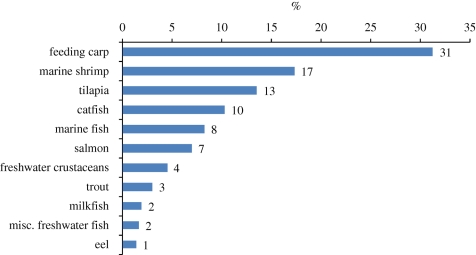
Estimated global compound aquafeed production in 2008 for major farmed species (as percentage of total aquafeed production, dry feed basis. Source: Tacon & Metian (in preparation).

**Figure 10.**
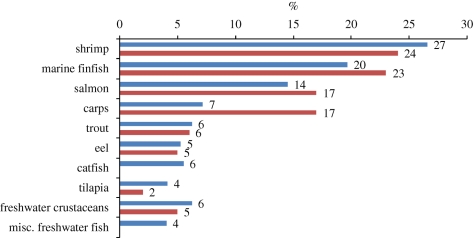
Estimated use of fish meal (percentage of dry feed basis) within aquafeeds in 2008. Blue, Tacon estimate 2008; red, IFFO estimate 2007. Sources: Tacon & Metian (in preparation); IFFO (2008).

**Figure 11.**
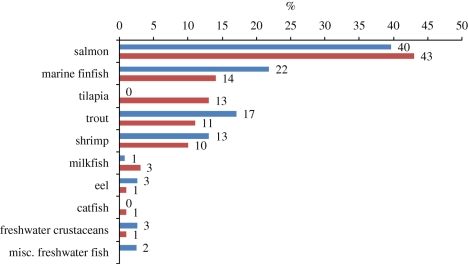
Estimated global use of fish oil (percentage of dry feed basis) in 2008. Blue, Tacon 2008; red, IFFO 2007. Sources: Tacon & Metian (in preparation); IFFO (2008).

**Figure 12.**
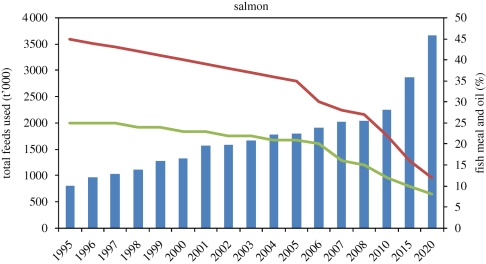
Estimated global use of fish meal and oil by the salmon farming industry projected to 2020. Blue, total feeds used; red, mean % fish meal; green, mean % fish oil. Source: Tacon & Metian (in preparation).

Wild-caught supplies of fish meal and oil have varied at around 5–6 million and 1 million tonnes annually for at least the past 20 years, suggesting that such levels are likely to be sustained in the future. However, in 2008, approximately 90 per cent of the fish oil available worldwide, and 71 per cent of the fish meal, was consumed in aquaculture practices (Tacon & Metian in preparation). Unless alternative higher value markets develop, aquaculture will continue to consume the majority of fish meal and oil produced but this will not be sufficient to meet ever-increasing demands for aquafeed ingredients (figures 9–12).

Feeds for herbivorous and omnivorous species (carps and tilapias) often contain fish meal and sometimes fish oil, although this is not essential on purely nutritional grounds. However, rapidly expanding culture of carnivorous species such as cobia and pangasius catfish could increase the pressure on fish meal and oil supplies. An overarching factor that has significantly impacted demands for fish meal and oil is improvements in food conversion efficiency as feeds and feeding technologies improve. Up to 25 per cent of fish meal is now obtained from fish processing waste, and ingredient substitution is also increasing the efficiency of fish meal and oil utilization. In the wild, the conversion efficiency (fish intake to fish output, FIFO) is commonly taken as 10 : 1 between one trophic level and the next (e.g. carnivorous fish eating plankton-feeding fish). Between 1995 and 2006, input : output ratios for salmon improved from 7.5 to 4.9, trout from 6.0 to 3.4, marine fish from 3.0 to 2.2 and shrimp from 1.9 to 1.4. Herbivorous and omnivorous finfish and some crustacean species showed net gains in output, with ratios in 2006 of 0.2 for non-filter feeding Chinese carp and milkfish, 0.4 for tilapia, 0.5 for catfish and 0.6 for freshwater crustaceans (Tacon & Metian 2008). Calculations of FIFO for the global aquaculture industry include 0.7 (Tacon & Metian 2008), 0.63 (Naylor *et al*. 2009) and 0.52 Jackson (2009). Overall, the finite supply of fish meal and oil is not expected to be a major constraint, but demand for alternative feed materials will increase—in turn placing greater pressure on the wider agro-feed system.

## 3. Addressing the challenges of aquaculture development

Looking forward, there is strong focus on improving the efficiency of resource utilization through management and integration or more technological solutions available through advances in engineering and bio-science. Both approaches will be important and influenced by wider social and economic factors including globalization, urbanization, factor prices (especially energy) and consumer demand.

### (a) Integration approaches

The integration of aquaculture, fisheries, agriculture and other productive or ecosystem management activities has an integral role to play in the future of the aquaculture industry. Techniques include ranching, agriculture/aquaculture (IAA), integrated multi-trophic aquaculture (IMTA) and links with renewable energy projects. Integration is a key element of the ‘ecosystem approach to aquaculture (EAA)’ which ‘is a strategy for the integration of the activity within the wider ecosystem in such a way that it promotes sustainable development, equity, and resilience of interlinked social and ecological systems’ (Soto *et al*. 2008).

#### (i) Integrating aquaculture and fisheries

Although aquaculture and capture fisheries are often seen as separate activities linked only in their market destinations, a number of important system linkages exist between these forms of aquatic production. These include interdependence for supplying fish products in aquaculture feeds, the role of aquaculture stocks in supporting and enhancing capture fisheries and the development of managed ecosystem approaches connecting aquaculture and fisheries in single spatial units; typically, lakes and floodplain systems, peri-urban zones, coastal margins and fjords or sea lochs.

Aquaculture-based fisheries enhancements comprise a diverse set of resource systems that combine attributes of aquaculture and fisheries. Most commonly, enhancements involve releases of cultured fish into open waters with the aim of enhancing fisheries catches directly or helping to rebuild depleted fish stocks. Examples include large-scale culture-based fisheries for major carps in Asian reservoirs, Pacific salmon ranching, scallop enhancements in Japan and New Zealand, and many systems that operate at smaller scales. Enhancements may also involve habitat and environmental modifications with the dual aim of increasing the productivity of wild or released cultured stocks and extending private ownership over such resources. Examples include traditional systems of culturing animals that recruit into privately owned and managed coastal ponds or rice fields and recent innovations such as ‘free fish farms at sea’ where fish are habituated to feeding stations.^2^

Major advances in the understanding of aquaculture-based enhancement fisheries systems and in underlying science areas have been made over the past decade. Integrative frameworks have been developed that allow a rapid assessment of enhancement potential based on the consideration of ecological, genetic, technological, economic, stakeholder and institutional attributes (Lorenzen 2008). Quantitative assessment tools can be used to evaluate the likely fisheries benefits of enhancements prior to and during the development of enhancement technologies (Lorenzen 2005; Medley & Lorenzen 2006). Robust genetic management principles have been defined for different types of aquaculture-based enhancements (Utter & Epifanio 2002). Understanding of domestication effects on fish behaviour has been applied to developing increasingly effective ways of conditioning cultured fish to improve their post-release survival and recapture (Olla *et al*. 1998). The economics of fisheries enhancements and, in particular, the institutional arrangements that can facilitate the emergence of such systems and sustain them over extended periods of time are now well understood (Arnason 2001; Lorenzen 2008).

Aquaculture-based fisheries enhancements can pose substantial ecological and genetic risks to wild fish stocks. In production-oriented enhancements, such risks can be minimized but not fully avoided by separating the released cultured and wild stocks ecologically (e.g. by release and habituation in habitats not used by interacting wild fish) and genetically (e.g. by maintaining captive brood stock and releasing sterile fish). Selective harvesting of released cultured fish may further reduce impact on wild stocks where this is technically possible (i.e. fishing is not unselective for cultured/wild fish). Environmental modifications and feeding could lead to further impacts on wild stocks and the natural ecosystem. In initiatives aimed at rebuilding wild stocks, the aim is for cultured fish to interact with wild fish and particular care must be taken in stock and genetic management to avoid detrimental impacts on the depleted or even endangered wild stock. Captive breeding and supplementation programmes can aid conservation and restoration of such stocks, but the management strategies in this case are very different from those employed in production-oriented enhancements.

Aquaculture-based fisheries enhancements are now successfully implemented in over 27 countries worldwide, involving over 80 species and yielding an estimated 2 million tonnes of fisheries products. It is therefore likely that interest in enhancements and demand for research and technology development in this area will increase.

#### (ii) Integrated multi-trophic aquaculture

IMTA systems can be described as culture systems that use species from different trophic levels grown in combination within the same water body or through some other water-based linkage (for land-based systems). Scale does not necessarily have to be large, provided the layout of the species being grown and the quantities being grown are compatible. In all cases water is the nutrient transport vector for dissolved and particulate wastes, the releases from one species acting as food for other species at a lower trophic level.

The combination of species from different trophic groups creates a synergistic relationship which, in turn, acts as a bioremediation measure. In a perfect IMTA system the processing of biological and chemical wastes by other species would make the whole production cycle environmentally neutral. There are IMTA systems at or near commercial scale in China, Chile, Canada, Ireland, South Africa and the UK, and ongoing research in many other countries. Such systems face a number of challenges, particularly in selecting species that integrate well, but that also have sufficient economic value to attract investment. The internalization of environmental costs within the systems (environmental economics) could substantially alter this (Soto 2009), as could the development of new products from marine species (Barrington *et al*. 2009). Other constraints include existing regulations restricting further aquaculture development or the potential for unintended interactions between systems. However, the potential of the approach in addressing sustainability objectives is clear.

#### (iii) Integrated aquaculture/agriculture

IAA is most common in developing countries, where it provides a means for rural systems to diversify and maximize output. The culture method differs from mono-culture, which is often too risk intensive for resource-poor farmers. Integrated systems benefit from the synergies among the different components and they have diversity in production that results in a relative environmental soundness (Prein 2002).

IAA systems range from simple integration to multi-component integrated systems using commercial fertilizers and feeds. Examples of IAA include the culture of fish in rice fields or the use of livestock manure from terrestrial farming for both feed and fertilizer in fish ponds. Integration can be categorized into: (i) polyculture (multiple species co-cultured; (ii) sequential (waste flows directed sequentially between culture units); (iii) temporal (replacement of species within the same holding site to benefit from waste generated by preceding species); and (iv) mangrove integration (using mangroves as biofilters) (Troell 2009).

Dey *et al*. (2010) evaluated the impact of a WorldFish-supported programme that introduced IAA to smallholders in Malawi and found adopters of the technology realized an 11 per cent rise in total factor productivity (TFP), 35 per cent higher technical efficiency scores, 134 per cent higher farm income per hectare and 60 per cent higher income overall compared with non-adopters. Non-adopters had higher income from off-farm activities, but adopters had higher overall returns to family labour and thus higher household incomes (almost 1.5 times higher). The authors suggest this illustrates the potential for IAA to contribute to poverty reduction and livelihood improvements in Malawi and probably other countries that have similar conditions.

Where IAA is practised on a larger scale and with commercial products, further challenges have emerged. For instance, quality can be variable with concerns about contamination, e.g. with pesticides where irrigation water is used, or off-flavour taints, which inhibit acceptance and certification, particularly for international markets (Little & Edwards 2003). To date, the benefits of IAA have focused mainly on food production, but more efficient use of freshwater and energy may become equally important.

In developed countries, there is growing interest and activity with small-scale aquaponic systems, which combine freshwater aquaculture in a recirculated system with hydroponic horticulture, usually herbs and salad vegetables. The horticultural crop is mostly fertilized by the nitrogen waste from fish culture. Owing to scaling issues, these systems have not proved attractive commercially, but are suitable for ‘back-yard’ food production, which is emerging as a candidate strategy for increasing sustainable food production.

### (b) Technical responses to resource issues

#### (i) Fish meal and fish oil replacement/substitution

Substitution of the protein (essential amino acids) and other nutrients derived from fish meal is nutritionally straightforward and considerable advances in this field have been made over the past 30 years. For protein supply, the issue is largely one of economics and formulation as well as continual assessment of potential novel sources of protein (such as: the biomass derived from bioethanol production; cereal glutens; microbial proteins; improved oilseed and legume meals, etc.). Even for carnivorous species (high dietary protein levels, sensitivity to the palatability of the feed), up to 75 per cent of the fish meal in a standard feed can easily be replaced (Bell & Waagbo 2008). For omnivorous and herbivorous species, fish meal is unnecessary and is only presently used because it is economically viable to do so.

There is a general issue of whether it is ethical, or impacts fish welfare, when carnivorous species are fed on ‘vegetarian’ diets. In addition, there is evidence that soya bean induces enteritis in Atlantic salmon and it is possible that plant proteins in general (which contain wide ranges of nutrient and non-nutrient fractions to which fish are not normally exposed) may have impacts on fish welfare.

Substitution of fish oil is considerably more problematic as *n* − 3 HUFA (highly unsaturated fatty acids; EPA and DHA) supplied by fish oil, and essential in the diets of truly marine species, are not commercially available from any other source at present. Neither is it desirable to reduce the *n* − 3 content of farmed species with respect to human health benefits. Considerable progress has therefore been made towards substitution of most or all of the fish oil during the growth phase before introducing a finishing diet, rich in fish oil, that ‘washes out’ the *n* − 6 fatty acids accumulated during growth. This results in a high *n* − 3 HUFA final product that resembles wild individuals of the same species.

For future supply of HUFA that can be incorporated in aquafeeds, some microorganisms (bacteria and algae particularly) have shown promise and HUFA yields will undoubtedly be increased through conventional selection, improved culture techniques and/or the use of genetically modified organisms. It may even be possible to combine production of useful protein biomass and HUFA in this way (Olsen *et al*. 2008).

One further potential source of feed protein and oil is krill (a collective name for a group of approximately 80 species of small, pelagic, shoaling crustacea). The nutritional issues of product quality (rapid spoilage) and fluorine content have been successfully addressed and viable methodologies for capture and processing developed. CCAMLR^3^ estimates a total allowable catch that would provide approximately 1 teratonne of krill meal and 32 000 million tonne of krill oil per year from Antarctic waters. However, aquaculture faces strong competition for the krill resource from increasing use of high grade krill for direct human consumption and production of pharmacological grade krill oils. The potential impact on marine food webs should also be seriously considered.

#### (ii) Genetic management and stock improvement

The bulk of aquaculture production still comes from wild or recently domesticated stocks. A lack of genetic management and poor hatchery procedures, particularly but not only in developing countries, has significantly degraded the performance of many farmed species through inbreeding, genetic drift and uncontrolled hybridization. The reduction in performance and viability means that hatchery stocks often need to be routinely replaced by wild fish or better managed stock from other farms. In contrast, properly managed selective breeding programmes have shown continual improvements in performance and quality. Atlantic salmon breeding companies have shown more than 100 per cent improvement in growth performance in around six generations, with significant improvements in disease resistance and delays in the onset of sexual maturation. The vast majority of farmed Atlantic salmon eggs and smolts are now sourced from such breeding companies and similar approaches are now being introduced in some other species.

Selective breeding can improve the year-on-year performance of farmed fish stocks for a wide range of traits, but it is still often necessary to include some other techniques that enable these fish to achieve their full potential. Sexual maturation in production fish can significantly reduce the final yield, as maturing fish can become aggressive, stop growing, lose condition and become more susceptible to disease. In many species one sex or another is preferred, e.g. because it grows faster or is still immature at harvest size. In salmonids, females usually mature later than males. In rainbow trout being grown to portion size (more than 300 g), all-female production is now almost universally used in Europe as females are still immature at harvest. In tilapia, all-male production is preferred: even though the males mature, the lack of females avoids the unwanted production of fry common in mixed sex on-growing systems.

In some species and under certain conditions, any sexual maturation is detrimental. This can be avoided by the production of sterile fish using chromosome set manipulation techniques that produce animals with three sets of chromosomes, known as induced triploidy. This approach is now used in the production of large rainbow trout (more than 3 kg) which continue to grow and remain in prime condition. Triploidy is also widely used for the production of ‘all-year-round’ oysters.

Transgenic technology has been applied to a number of fish species in recent years, although mostly for research. Recent studies in salmonids show that the spectacular improvements in growth seen by incorporating growth hormone gene constructs into slow-growing wild strains were not repeated when the same constructs were incorporated into fast-growing domesticated stock (Devlin *et al*. 2009). This suggests that the same improvement in growth could be achieved using selective breeding techniques which have the advantage of selecting across a range of commercial traits, raising the overall performance of the strain as well as maintaining its genetic integrity. Transgenic strains are by necessity derived from a small number of individuals, making further improvement in other commercial traits less likely. In the EU, the high level of public concern about GM technology would suggest that the widespread adoption of transgenic fish for a single trait such as growth performance, even if it were licensed, would meet with consumer resistance.

#### (iii) Welfare and health management

Disease has proved a major constraint to efficient production in some intensive aquaculture systems. Major improvements in the understanding of the aetiology and epidemiology of fish diseases have been made in recent years and aquaculture producers in many countries have dramatically improved their husbandry practices with greater focus now on fish welfare. Control of many serious infectious diseases has been achieved through new medicines and vaccines, and this is especially true for bacterial diseases. However, new disease problems are emerging, and previously rare diseases becoming much more prevalent, so continued vigilance and solution development is required.

Vaccines have been very effective for bacterial fish pathogens where there are resources to develop them, but success against virus disease has been more limited. Nevertheless, fish viral diseases were among the first to be tackled using recombinant DNA technology, specifically for infectious pancreatic necrosis, and subsequently direct DNA vaccination, which appears very promising. As this involves a transfer of genes, there are significant issues of safety and consumer acceptance to be addressed. Another approach showing promise is the use of proteomics and epitope mapping for the identification of vaccine antigens and the subsequent development of peptide vaccines. It is hoped that this approach might be suitable against parasites such as salmon lice. Further methods include the use of virus-like particles which have been reportedly used against grouper nervous necrosis virus or recombinant viral proteins produced in yeast (Renault 2009).

For the moment, new therapies developed using genomic tools appear some way off, but some potential has been demonstrated using dsRNA for disease protection and RNA-i-based gene therapies in shrimp (Renault 2009). Antimicrobial peptides are also being studied as a potential therapeutant. Aquaculture diets are also under scrutiny with respect to potential for delivery of immunostimulants and better understanding of interactions between gut microflora, pathogens and micronutrients, including probiotic effects (Gatesoupe 2009).

#### (iv) Engineering and systems technology

With respect to the engineering of culture systems, aquaculture largely takes and adapts technology from other sectors, such as fisheries, water treatment or offshore oil. However, as the sector grows, more specialized equipment develops, such as the well boats now currently employed by the salmon industry. Of particular interest for reducing pressure on water resources and minimizing impacts on sensitive freshwater or coastal environments, are recirculated aquaculture systems (RAS) and offshore cage technology.

#### (v) Recirculating aquaculture systems

RAS culture systems are typically land-based, using containment systems such as tanks or raceways for the fish. A percentage of the water is passed from the outflow back through the system following treatment and removal of wastes. The level of waste treatment and water reuse depends largely on the requirements of the fish, the environmental parameters and the technology available. Reusing water gives the farmer a greater degree of control over the environment, reduces water consumption and waste discharge and enables production close to markets (Sturrock *et al*. 2008). Owing to relatively high capital costs, high energy dependencies and more complex technology, RAS is largely restricted in its use to higher value species or life stages (especially hatcheries where control over environmental conditions is more critical and unit values higher). However, it could become a more competitive approach if economic factors change.

#### (vi) Offshore cages

Moving systems further offshore removes a number of the challenges faced by near shore systems such as visual impacts, local environmental impacts and space constraints. In most cases, predation issues and disease risks could also be substantially reduced. Expansion of the offshore industry would allow increases in the scale of project and could therefore improve efficiency as well. Competition with other interests such as tourism and inshore fisheries might be reduced and waste discharges would be more readily diffused. However, other problems and risks associated with intensive cage-based aquaculture would remain or even increase.

There is no internationally agreed definition of offshore cage aquaculture. In Norway, sites are classified according to significant wave height, whereas in the USA offshore aquaculture is defined as operations in the exclusive economic zone from the three mile territorial limit of the coast to 200 miles offshore (James & Slaski 2006). In general, offshore farming can be characterized as more than 2 km from shore, subject to large oceanic swells, variable winds, reduced physical accessibility and requiring mostly remote operations including automated feeding and distance monitoring. For these reasons, offshore aquaculture systems need to be robust structures and associated systems which are able to function with minimum intervention in a high-energy environment (Sturrock *et al*. 2008). There are also substantial issues over staff safety which increase cost over near shore systems.

The large size required and amount of new technology mean that offshore cage farms will have large capital requirements, which will restrict use until farms and companies reach a scale of operations where offshore investment becomes feasible. There are signs that this is starting to happen with Marine Harvest, the largest salmon farming company, which has announced an intention to apply for and develop offshore sites. This is for salmon farming, but several species have been promoted as potentially suitable for offshore farms (on the basis of biology and economics), with cobia perhaps receiving the most interest and investment.

#### (vii) Information technology

Advances in information and communications technology is benefiting the aquaculture industry with improved monitoring and control systems and better real-time information for managers. The development of micro-sensors combined with greater sophistication in electronic tags is opening up possibilities for data collection from individual fish within an aquaculture environment. Particularly, when combined with genomic tools, this is a potentially powerful research approach and may also play a role in management feedback (Bostock 2009). A notable development in the British trout sector is the linking of data from many farms to provide both a benchmarking tool for farm management and stock performance, and data for real-time epidemiological modelling. This is based on changes in mortality patterns reported by the farm and their geographical location and basic environmental data such as water temperatures. Such tools can potentially provide early warning of disease outbreaks in the industry and allow precautionary actions to be put in place.

## 4. Conclusions

All forward projections anticipate a need for increased supply of fish protein to meet the health needs and general aspirations of societies. Furthermore, this will need to be at affordable levels in relation to income and other proteins. As with terrestrial animal proteins, production of fish protein is more ecologically expensive than production of plant protein due to the higher trophic level, although some systems (e.g. enriched polyculture ponds) compare very well. Bivalve shellfish should also not be overlooked as an animal protein already well ahead on sustainability criteria.

With respect to fisheries and aquaculture, it may be helpful to break the market down into commodity products that are used in a wide range of food presentations and outlets (such as whitefish, salmon, tuna and prawns), and products that are differentiated through distinctive attributes and that have both smaller production and market bases. Bulk supply is most likely to come through growth in the globalized commodity products based on economies of scale, while growth in the more specialist products would be through diversification of products and production systems.

Underlying development of sustainable aquaculture of all types, but especially commodity products, is the need to improve the basic conversion of feed materials into edible fish flesh and minimize utilization and conversion of premium resource. This involves species selection, production systems, animal genetics, good health management and optimized feed and feeding. These are also linked to some extent through the developing understanding of animal welfare, which is also reaching into other physiological and environmental interactions. The interactions of aquaculture with the environment, with respect to both goods and services, are also critical and need to be evaluated in a rational way that allows the benefit of environmental services to be used but not over-exploited and impacted on.

At the policy level, important questions exist about the priority given to conserving the environment versus the exploitation of natural resources for food production. While richer nations in Europe may be able to offset reduced food production by increasing imports, the environmental impact is transferred to other countries where options or control are more limited. Imposing high environmental standards on both local production and imports would encourage technology development and uptake, although most likely at the cost of increased food prices.

With the market of central importance to the direction of future development, there is growing momentum to educate and influence market demand to play a more responsible role in shaping future production systems. Many campaign groups are active on specific issues, which is at least stimulating debate and further developments. Most notably, there is now a clear trend towards the establishment of various types of standards that can be measured, monitored and certificated by independent bodies to provide producers with clear guidelines and consumers and market chain participants with confidence in the environmental or social provenance of the product. The development of appropriate standards can, however, be challenging. Within aquaculture, there are now many initiatives, perhaps most significantly GLOBALGAP,^4^ which is private sector-based business-to-business certification focusing on food safety, animal welfare, environmental protection and social risk assessment standards. This now has certification schemes for shrimp, salmon, pangasius and tilapia and is developing a standard for aquaculture feeds. While GLOBALGAP has strong take-up, it does not involve a specific consumer label, such as ‘Friend of the Sea’,^5^ ‘Freedom Foods’^6^ or various organic labels. So far, aquaculture products have not had a consumer label with the degree of recognition of the Marine Stewardship Council mark for sustainable capture fisheries. This is expected to change with the formation of the Aquaculture Stewardship Council^7^ which is taking forward a long programme of stakeholder dialogues organized by the WWF^8^ on standards for 12 major aquaculture products and implementing a consumer-oriented certification scheme.

The WWF aquaculture dialogues have highlighted the problems in developing robust measures of sustainability, particularly as definitions move beyond simple measures of environmental impact to more complex assessments of ecological efficiency. Parallel initiatives by international policy and academic organizations have therefore focused on the development of assessment tools. LCA is one of the key approaches, measuring parameters such as total energy consumption or carbon emissions throughout the production, distribution, consumption or disposal of individual products. This allows a ready comparison between products and helps to identify stages in the product life cycle where efficiency gains might be realized. While LCA provides a useful headline figure, it is less useful for understanding the dependencies of products on natural resources and service or linkages to other production processes. For this reason, FAO and partners are developing assessment frameworks based on the concept of an EAA^9^. This uses a number of measures including the concept of ecological footprints which help assess the dependence of specific activities on ecosystem support. A further tool that may prove useful is the ‘Global Aquaculture Performance Index’^10^ developed by the University of Victoria, Canada, and based on the Yale and Columbia University's Environmental Performance Index^11^. This uses a range of weighted metrics and statistical analysis to provide comparative scores for assessing species choices or performance differences between countries or regions.

While the creation and use of international standards may appear an irrelevance to smallholder systems in many countries, there is also a risk that they could create substantial barriers to development, by denying them access to wider markets. The implications of globalizing trade, standards and certification, development and sustainability and how these interrelate are being researched by the EC-funded SEAT project,^12^ which aims to build a broader scoring system encompassing a range of ethical issues.

Future policy development will clearly need to move beyond simple objectives of economic development and employment or environmental protection and conservation. The complexity of the seafood market suggests there are many opportunities for segmentation and innovative approaches to sustainable aquaculture that could be exploited with policy support.

## End notes

1 In 2008, brackish-water aquaculture produced 4.1 million tonnes valued at US$13.1 billion; shrimp and prawn production was 2.4 million tonnes valued at US$9.4 billion.

2 http://www.atc.stir.ac.uk/FFFS/Bg.html.

3 http://www.ccamlr.org.

4 http://www.globalgap.org.

5 http://www.friendofthesea.org.

6 http://www.rspca.org.uk/freedomfood.

7 http://www.ascworldwide.org.

8 http://www.worldwildlife.org/what/globalmarkets/aquaculture/aquaculturedialogues.html.

9 http://www.fao.org/fishery/topic/16035/en.

10 http://web.uvic.ca/~gapi.

11 http://epi.yale.edu/Home.

12 http://seatglobal.eu.
